# A Quantitative Estimate of the Expected Shortening of the Median Isolation Period of Patients With COVID-19 After the Adoption of a Symptom-Based Strategy

**DOI:** 10.3389/fpubh.2021.639347

**Published:** 2021-06-10

**Authors:** Francesca Bai, Alessandro Tavelli, Giovanni Mulè, Camilla Falcinella, Debora Mondatore, Daniele Tesoro, Diletta Barbanotti, Daniele Tomasoni, Roberto Castoldi, Matteo Augello, Marina Allegrini, Gianmarco Tagliaferri, Andrea Cona, Alessandro Cozzi-Lepri, Giulia Marchetti, Antonella d'Arminio Monforte

**Affiliations:** ^1^Department of Health Sciences, Clinic of Infectious Diseases, San Paolo Hospital, ASST Santi Paolo e Carlo, University of Milan, Milan, Italy; ^2^Research Department of Infection and Population Health, Institute for Global Health, Centre for Clinical Research, Epidemiology, Modelling and Evaluation, University College London, London, United Kingdom

**Keywords:** COVID-19, SARS CoV-2, molecular diagnosis, isolation and quarantine, criteria for releasing COVID-19 patients from isolation

## Abstract

A long period of isolation was observed in patients hospitalized for COVID-19 in Milan over March-September 2020 (45; IQR: 37–54 days). A significantly shorter period would have been observed by the application of May-WHO (22, IQR: 17–30 days, *P* < 0.001) and October-Italian (26, IQR: 21–34 days, *P* < 0.001) Guidelines. The adoption of the new symptom-based criteria is likely to lead to a significant reduction in the length of the isolation period with potential social, economic and psychological benefits, particularly in the younger population with mild/moderate disease and no comorbidities. In our opinion, the release from isolation after 21 days from symptoms onset, even without a PCR diagnostic test, in most cases seems the most adequate strategy that could balance precautions to prevent SARS CoV-2 transmission and unnecessary prolonged isolation or overuse of diagnostic testing.

## Introduction

Accumulating data show that a replication competent SARS CoV-2 virus is rarely found in respiratory samples after 9–10 days from symptoms onset, while rRT-PCR on oro- or naso-pharyngeal swabs may remain persistently positive up to 3 months from the onset of SARS CoV-2 infection (1, 2).

Furthermore, worldwide cases of SARS CoV-2 RNA turning to positive, with or without recurrent symptoms after clinical recovery, are not associated with the isolation of competent virus in culture in most cases (3–5). As a consequence, WHO recommendations to release COVID-19 patients from isolation changed overtime, according to new evidence, as well as other International and Italian Guidelines (6–8) (Table 1).

**Table 1:** Criteria for releasing COVID-19 patients from isolation.

**Old criteria**	**Updated criteria**
**WHO, 12 January 2020**	**WHO, 27 May 2020**
Clinical recovery and two negative RT-PCR results on sequential samples taken at least 24 hours apart.	10 days after symptom onset and at least 3 additional days without symptoms (including without fever and respiratory symptoms)
**Italian Ministry of Health, 28 February 2020**	**Italian Ministry of Health, 12 October 2020**
After clinical recovery: Two consecutive negative SARS-CoV-2 RT-PCR tests in a 24-h interval from respiratory specimens.	One negative SARS-CoV-2 RT-PCR test from respiratory specimens after 10 days from symptom onset including at least 3 days without symptoms Persistent RNA positive patients: 21 days from symptom onset (without repeating SARS-CoV-2 RT-PCR test) and at least 7 days without symptoms

In this analysis, we calculated the median isolation period using real data of COVID-19 patients admitted to two tertiary hospitals in Milan over the period March-September 2020 and we provided an estimate of the shortening of this time under the hypothetical scenario of an isolation period as recommended by the current WHO and October-Italian guidelines (6, 7). We aimed to predict the median isolation period for people with similar characteristics during the second wave of epidemic in Milan and to identify the patients who are likely to most benefit from the reduction of their isolation period.

## Methods

### Study Population

In this prospective observational study we included patients fulfilling the following criteria:

- hospitalized for COVID-19 symptomatic infection (from March 1st to September 30th, 2020) at San Paolo and San Carlo hospital, Milan, Italy;m
- discharged from hospital with clinical recovery (apyrexia from ≥72 h and normal respiratory rate in room air);
- two negative rT-PCR for SARS CoV-2 (ELITeInGenius®system and the GeneFinder COVID-19 Plus RealAmp Kitassay; ELITechGroup, France) on naso-pharingeal swabs, according to the February-Italian Guidelines (9).

After hospital discharge, patients were followed-up in an outpatient service to monitor the virological clearance. Naso-pharingeal swabs were repeated every 7 days till two consecutive negative tests. Patients who obtained two RNA negative swabs were given a certificate of virological recovery, attesting end of isolation.

Patients who performed nasopharyngeal swabs to document virological clearance outside our outpatient services and for whom data of end of isolation was unknown were excluded from the analyses.

We considered the following patients' characteristics: age (<50, 50–69, and ≥70 years), Charlson comorbidity index (CCI) (score 0, 1, 2, and ≥3) (10) and the maximum grade of respiratory support, as proxy of disease severity: no O_2_ therapy (mild disease); low/high O_2_ flows (moderate disease); Continuous Positive Airway Pressure (cPAP) (severe disease); Non Invasive Ventilation (NIV); and Invasive Mechanical Ventilation (IMV) (critical disease).

### Estimation of the Median Time From Symptoms Onset to Release From Isolation Under Three Possible Scenarios

We calculated the median time from symptoms onset to release from isolation under three scenarios: (i) the factual scenario (what has actually happened in March–September 2020); (ii) counterfactual scenario A: if the May-WHO criteria were adopted in March (7); (iii) counterfactual scenario B: if the October-Italian criteria (6) were adopted in March.

Median time to end of isolation under the three scenarios was determined for the whole cohort and compared using non parametric Wilcoxon test for paired data. Mean (±standard deviation, SD) of isolation time was also calculated for specific subgroups (according to age, CCI, and disease severity). We calculated marginal means of estimated time spent in isolation under the two counterfactual scenarios and the average treatment effect with bootsrap 95% Confidence Intervals (CI) using the factual scenario as the comparator.

We classified participants according to whether the isolation time under the WHO scenario was >20 days shorter than the actual time. This threshold was chosen under the assumption that 20 days of shorter isolation was enough to have a significant impact on quality of life and utilization of health resources.

We then calculated marginal probabilities by fitting a logistic regression using age strata (<50, 50–69, and ≥70 years), CCI (score 0, 1, 2, and ≥3) and disease severity (no O_2_ therapy; low/high O_2_ flows; cPAP; NIV; and IMV) as covariates without interactions and estimated the probability of a shortening of time spent in isolation by more than 20 days according to participants profiles; marginal plots by subgroups were shown.

The same logistic regression model has been used to calculate crude and adjusted odds ratios (OR/AdjOR) of a shortening of more than 20 days with 95% CI for the three variables (age, CCI, and disease severity); AdjOR were corrected for all the three variables included in the model. All analyses were performed using Stata (version 14, StataCorp, USA).

Informed consent from study participants was obtained; the study was approved by Ethic Committee-Area 1, Milan (2020/ST/049-2020/ST/049_BIS, 11/03/2020).

## Results

Four hundred and thirty patients were discharged from March 1st to September 30th, 2020 and kept in isolation until virological clearance.

Table 2 shows demographic and clinical characteristics of the study population. Median age was 59 years (IQR: 50–71) and 268 (62.3%) were males. Fifty-four (12.6%) and 109 (25.3%) received IMV/NIV or CPAP as highest grade of respiratory support during hospitalization, respectively. Median days from symptoms onset to clinical recovery were 19 (IQR: 14–27) and median length of hospitalization was 12 (IQR: 7–21) days (Table 2).

**Table 2:** Characteristics of study population.

**Characteristics**	**Study population (*N* = 430)**
**Age, years, median (IQR)**	59 (50–71)
**Age**, ***n*** **(%)**	
<50 years	104 (24.1%)
50–69 years	203 (47.1%)
≥70 years	123 (28.6%)
**Gender, males**, ***n*** **(%)**	268 (62.3%)
**BMI** **>30**, ***n*** **(%)**	64 (14.9%)
**Italian**, ***n*** **(%)**	324 (75.3%)
**Age-unadjusted Charlson score, median (IQR)**	0 (0–1)
**Age-unadjusted Charlson score**, ***n*** **(%)**	
0	283 (65.8%)
1	80 (18.6%)
2	33 (7.68%)
≥3	34 (7.92%)
**Symptoms at hospital admission**, ***n*** **(%)**	
Anosmia/dysgeusia	27 (6.3%)
Arthromyalgia	26 (6.1%)
Chest pain	18 (4.2%)
Cough	265 (61.6%)
Dyspnea	226 (52.6%)
Fatigue	79 (18.4%)
Fever	372 (86.5%)
Gastro-intestinal symptoms	82 (19.1%)
**Highest grade of respiratory support during hospitalization**, ***n*** **(%)**	
IMV or NIV	54 (12.6%)
CPAP	109 (25.3%)
O_2_ low/high flows	185 (43.0%)
No O_2_ therapy	82 (19.1%)
**Blood exams at hospital admission, median (IQR)**	
C Reactive Protein, mg/L	45.6 (18.7–88.3)
Lactate dehydrogenase (LDH), U/L	286 (222–364)
Lymphocytes, cells/mmc	1.070 (760–1460)
Hemoglobin, g/dL	13.7 (12.5–14.8)
Creatinine, mg/dL	0.9 (0.7–1.1)
**Days from symptoms onset to clinical recovery, median (IQR)**	19 (14–27)
**Days of hospitalization, median (IQR)**	12 (7–21)

*Quantitative data are presented as median (Interquartile Range), categorical data as absolute numbers (percentages). IMV, Invasive mechanical ventilation; NIV, Non Invasive Ventilation; CPAP, Continuous Positive Airway Pressure; Immunomodulating drugs, IL-6 receptors antagonists and JAK inhibitors*.

Median time from symptoms onset to isolation release was 45 (IQR: 37–54) days; median time from clinical recovery to isolation release was 23 (IQR: 19–31) days.

**Figure 1:**
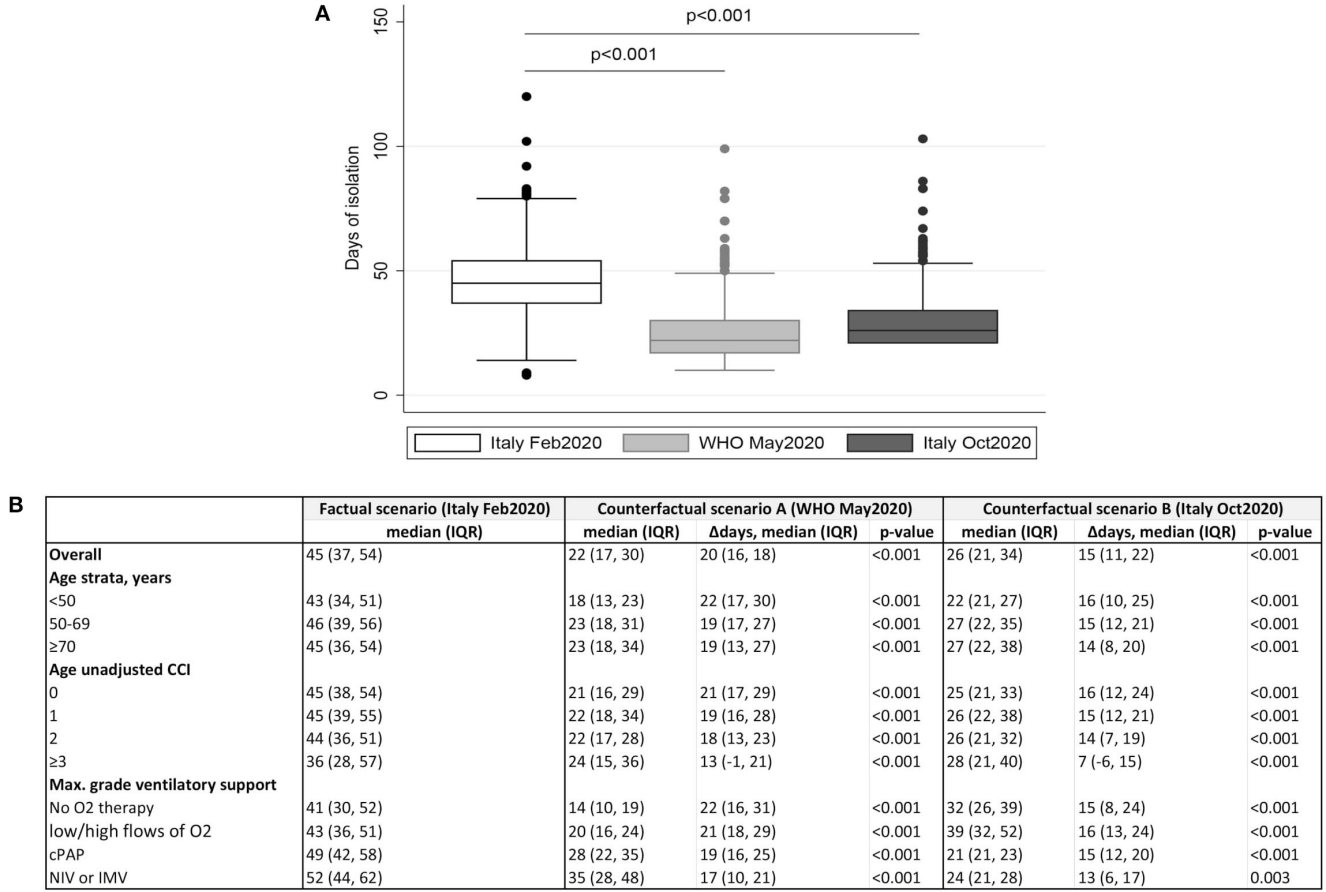
Comparison of time from symptoms onset to release from isolation by application of February-Italian, May-WHO, and October-Italian criteria. **(A)** Box plot representing median days spent in isolation by application of Italian and WHO criteria for releasing COVID-19 patients from isolation. *P*-values by Wilcoxon signed rank test for paired data. **(B)** Mean (±standard deviation) of the isolation time under the three scenarios (February-Italian, May-WHO, and October-Italian guidelines) according to age, CCI, and maximum grade of respiratory support. Average treatment effects (95%CI) for WHO May 2020 and Italian October 2020 criteria, using the factual scenario (Italian February 2020) as the comparator, were shown.

A shorter time would have been observed by the application of the May-WHO (22, IQR: 17–30 days, *P* < 0.001) and the 12 October-Italian criteria (26, IQR: 21–34, *P* < 0.001; Figure 1A). The estimate using WHO counterfactual scenario A was significantly shorter also compared to scenario B (*P* < 0.001; Figure 1A).

The estimated mean days of isolation in the three scenarios according to age, CCI, and severity of the diseases are shown in Figure 1B. A significant reduction of isolation could have been occurred, regardless of patient's characteristics, under both counterfactual scenarios.

**Figure 2:**
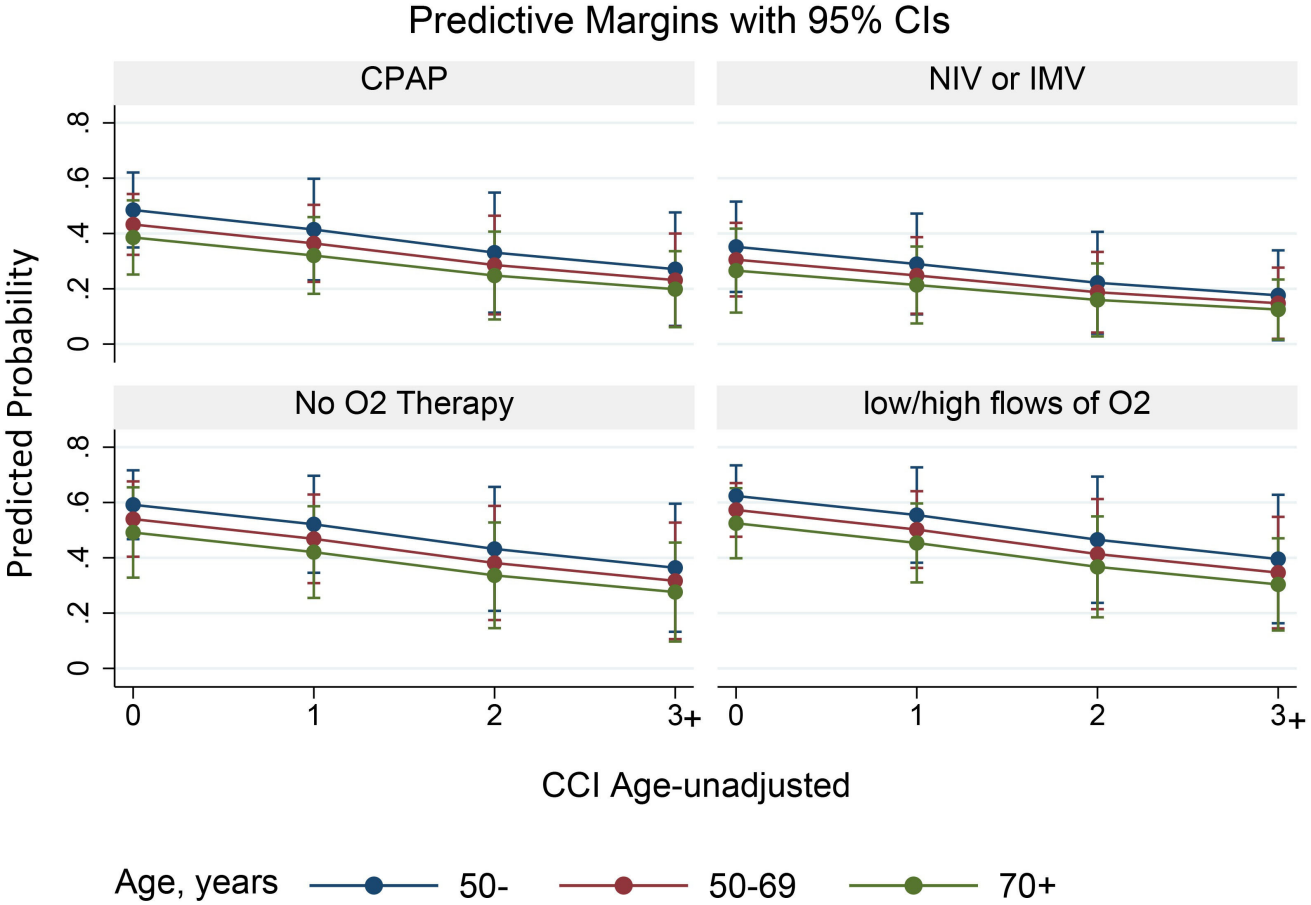
Plots of the marginal predictions of shortening the time spent in isolation by more than 20 days. Predictive margins with 95% CI by subgroups (age classes, CCI, and O_2_ therapy) are represented.

Nevertheless, some small differences have been detected; the estimated probability of observing a reduction of time spent in isolation by more than 20 days under the WHO scenario, compared to the actual scenario, was the highest in patients <50 years, without significant comorbidities (CCI = 0) and mild disease severity (low/high O_2_ flow; Figure 2). Patients aged >70 years old, with CCI ≥ 3 and severe disease were the group with the least estimated benefit (Figure 2).

By fitting a univariable logistic regression analysis, a higher probability of shortening the time spent in isolation by more than 20 days under the adoption of May-WHO criteria (counterfactual scenario A) compared to actual scenario were younger age (<50 vs. ≥70 years, OR = 2.04, 95%CI: 1.2–3.47, and *P* = 0.009), no comorbidities (age-unadjusted CCI = 0 vs. ≥3, OR = 2.84, 95%CI: 1.28–6.29, and *P* = 0.01) and mild severity (no O_2_ therapy vs. NIV/IMV, OR = 2.73, 95%CI: 1.31–5.7, and *P* = 0.008; low/high flows of O_2_ therapy vs. NIV/IMV, OR = 2.80, 95%CI: 1.45–5.44, and *P* = 0.002; Table 3).

**Table 3:** Factors associated with the reduction of time spent in isolation by more than 20 days under 27-may WHO criteria.

**Parameters**	***N* (%)**	**OR**	**95%CI**	***p*-value**	AOR^*^	**95%CI**	***p*-value**
**Age strata, years**							
≥70	(*N* = 123, 28.6%)	1			1		
50–69	(*N* = 203, 47.1%)	1.31	0.83–2.07	0.242	1.21	0.73–2.01	0.452
<50	(*N* = 104, 24.1%)	2.04	1.20–3.47	0.009	1.50	0.81–2.77	0.196
**Age unadjusted Charlson comorbidity index**							
≥3	(*N* = 34, 7.92%)	1			1		
2	(*N* = 33, 7.68%)	1.39	0.49–3.97	0.54	1.33	0.46–3.88	0.601
1	(*N* = 80, 18.6%)	1.95	0.81–4.71	0.138	1.90	0.76–4.76	0.169
0	(*N* = 283, 65.8%)	2.84	1.28–6.29	0.01	2.53	1.06–6.04	0.036
**Max grade of respiratory support**							
NIV or IMV	(*N* = 54, 12.6%)	1			1		
cPAP	(*N* = 109, 25.3%)	1.69	0.83–3.44	0.145	1.74	0.84–3.57	0.134
Low/high flows of O_2_ therapy	(*N* = 185, 43.0%)	2.80	1.45–5.44	0.002	3.05	1.55–6.03	0.001
No O_2_ therapy	(*N* = 82, 19.1%)	2.73	1.31–5.70	0.008	2.67	1.25–5.72	0.012

* *Adjusted for all the factors shown in the table*.

*Univariable and multivariable logistic regression analysis. OR, odds ratio; AOR, adjusted odds ratio; 95%CI, 95% confidence interval; IMV, Invasive mechanical ventilation; NIV, Non Invasive Ventilation; cPAP, Continuous Positive Airway Pressure*.

A medical history without significant comorbidities (age-unadjusted CCI = 0 vs. ≥3, AOR = 2.53, 95%CI: 1.06–6.04, and *P* = 0.036) and lower grades of respiratory support during hospitalization (low/high O_2_ flows vs. NIV/IMV, AOR = 3.05, 95%CI: 1.55–6.03, and *P* = 0.001 and no O_2_ therapy vs. NIV/IMV, AOR = 2.67, 95%CI: 1.25–5.72, and *P* = 0.012) were confirmed independently associated with a higher probability of reducing time in isolation by at least 20 days in the multivariable analysis (mutually adjusting for age, CCI, and severity of disease; Table 3).

## Discussion

With a view to the second wave of COVID-19 epidemic in Milan, our study suggests that the application of the recent and less restrictive Guidelines for releasing COVID-19 patients from isolation will result in a significant reduction of time spent in isolation in our setting.

Under the two counterfactuals scenarios (6, 7), a median of 15–20 days of isolation would be saved compared to the Italian criteria of February 2020. In fact, before 12 October 2020, the virological recovery, corresponding to the end of isolation, was defined only in case of two consecutive SARS-CoV-2 RNA negative swabs, taken 24–48 h apart, after clinical recovery (9); adopting this strategy, isolation period in March-September 2020 resulted extremely long as a substantial proportion of patients was persistently positive and went on repeating the nasopharyngeal swabs each week until reaching the virological clearance, in some cases months later.

Fear of transmission and unknown contagiousness period were the main determinants of these early recommendations resulting in prolonged isolation.

The consequences of retained isolation are both social and psychological (11). Considering that 52% of our patients is aged 60 or younger, the impact of prolonged isolation on their ability to keep their job can be dramatic.

Further, consistently with other studies (12, 13) we previously demonstrated that 30% of patients recovered from COVID-19 showed anxiety symptoms and had abnormal scores in the Hospital Anxiety and Depression Scale (HADS) 1–3 months after recovery (14).

Assuming that clinical characteristics of hospitalized COVID-19 patients in the first and second wave of the epidemic were comparable, the median isolation period for those patients can be estimated between 22 (17–30) and 26 (11–22) days. The lower estimate is calculated using the WHO counterfactual scenario A, which corresponds exactly to the best-case scenario of the new Italian guideline of October 2020 (negative SARS CoV-2 swab 10 days after symptoms onsets, 3 of which without symptoms). The highest estimate corresponds to the counterfactual scenario B, the worst-case scenario according to new Italian guidelines, for patients with persistent long-term SARS CoV-2 RNA positivity. This would amount to a shortening of the isolation period by a significant 15–20 days.

By adopting the new recommendations for releasing patients from isolation, the isolation period would shorten especially for patients without comorbidities and diagnosed with a not severe disease; in the first wave of epidemic in Milan also young subjects with mild disease who obtained early clinical recovery remained in home isolation for a long period pending virological clearance.

Other advantages of the shortening of the isolation period in hospital is the reduced burden on national health resources and the greater availability of extra space for people with acute disease who need urgent care.

Considering the growing evidence that, after 10 days following symptoms onset, rRT-PCR on upper respiratory samples could remain positive, but no replication-competent virus is recovered in viral cultures (1, 2, 15, 16), a test-based strategy appears to be inadequate at the current time.

However, a minimal residual risk of transmission exists when adopting the new criteria, as in few cases of severe COVID-19 disease and immunosuppression, competent virus, and thus contagiousness, has been reported till 20 days from symptoms onset (17). Furthermore, there might be situations in which this minimal risk is not acceptable (e.g., if a PCR-positive patient needs to be transferred into COVID-negative department together with immunocompromised/vulnerable patients). In these situations a laboratory-based approach can still be useful.

Finally, worldwide cases of recurrent symptoms after clinical recovery are scarce and in most cases are not associated with the isolation of competent virus in culture, but with a persistent positive RNA (3–5).

Possible limitations of our study are: (i) the lack of actual data on other cohorts that adopted WHO criteria for releasing patients from isolation for comparisons with our study population; in fact, we simulated the isolation time we would have had on our cohort of patients by adopting WHO and October 2020 Italian criteria. However, this approach has the advantage of better control for confounding in the logistic regression analysis as characteristics of patients in different pandemic waves can be dramatically different and this could bias the comparison; (ii) limited generalizability of the results to patients actually enrolled in subsequent waves as these might differ for key effect measure modifiers; (iii) missing data about health and financial outcomes associated with the reduction of time spent in home isolation; (iv) our data are related to a particular time-period of the COVID-19 pandemic and it might need to be adjusted as new variants of concern might arise in the future.

In conclusion, the use of a test-based strategy during the first wave of the pandemic in all COVID-19 patients, including young and mildly ill patients, led to long periods of hospital and home isolation with consequent economic and psychological damage. More and more data report the absence of contagiousness after 10 days following onset of symptoms, making symptoms-based criteria the most appropriate strategy currently. In our opinion a symptom-based strategy will lead to significant benefits in terms of quality of life and optimization of resources with little consequences in terms of risk of transmission, which should be however monitored.

## Data Availability Statement

The raw data supporting the conclusions of this article will be made available by the authors, without undue reservation.

## Ethics Statement

The studies involving human participants were reviewed and approved by Ethic Committee-Area 1, Milan (2020/ST/049-2020/ST/049_BIS, 11/03/2020). The patients/participants provided their written informed consent to participate in this study.

## Author Contributions

Ad'A, AC-L, FB, and AT developed the concept of this study. FB, AT, GM, CF, MAu, DB, DM, DTe, MAl, GT, and DTo collected data on Case Report Form and performed data entry. AT did the statistical analyses. FB wrote the manuscript. FB, AT, AC-L, GMa, and Ad'A contributed to the final text. All the authors revised the text critically and have read and approved the final text.

## Conflict of Interest

The authors declare that the research was conducted in the absence of any commercial or financial relationships that could be construed as a potential conflict of interest.
